# The Mixed Instrumental Controller: Using Value of Information to Combine Habitual Choice and Mental Simulation

**DOI:** 10.3389/fpsyg.2013.00092

**Published:** 2013-03-04

**Authors:** Giovanni Pezzulo, Francesco Rigoli, Fabian Chersi

**Affiliations:** ^1^Istituto di Linguistica Computazionale, “Antonio Zampolli,” Consiglio Nazionale delle RicerchePisa, Italy; ^2^Istituto di Scienze e Tecnologie della Cognizione, Consiglio Nazionale delle RicercheRoma, Italy

**Keywords:** model-based reinforcement learning, hippocampus, ventral striatum, goal-directed decision-making, exploration-exploitation, value of information, forward sweeps

## Abstract

Instrumental behavior depends on both goal-directed and habitual mechanisms of choice. Normative views cast these mechanisms in terms of model-free and model-based methods of reinforcement learning, respectively. An influential proposal hypothesizes that model-free and model-based mechanisms coexist and compete in the brain according to their relative uncertainty. In this paper we propose a novel view in which a single Mixed Instrumental Controller produces both goal-directed and habitual behavior by flexibly balancing and combining model-based and model-free computations. The Mixed Instrumental Controller performs a cost-benefits analysis to decide whether to chose an action immediately based on the available “cached” value of actions (linked to model-free mechanisms) or to improve value estimation by mentally simulating the expected outcome values (linked to model-based mechanisms). Since mental simulation entails cognitive effort and increases the reward delay, it is activated only when the associated “Value of Information” exceeds its costs. The model proposes a method to compute the Value of Information, based on the uncertainty of action values and on the distance of alternative cached action values. Overall, the model by default chooses on the basis of lighter model-free estimates, and integrates them with costly model-based predictions only when useful. Mental simulation uses a sampling method to produce reward expectancies, which are used to update the cached value of one or more actions; in turn, this updated value is used for the choice. The key predictions of the model are tested in different settings of a double T-maze scenario. Results are discussed in relation with neurobiological evidence on the hippocampus – ventral striatum circuit in rodents, which has been linked to goal-directed spatial navigation.

## 1 Introduction

Goal-directed decision-making describes choice as depending on the evaluation of action-outcome contingencies (Balleine and Dickinson, 1998). Consider the case of a thirsty rat facing a T-maze with water in its left end. When behavior is controlled by goal-directed mechanisms of choice, the rat goes left because it predicts a water outcome (expectancy), and wants to reach it (goal state). Goal-directed mechanisms are considered to be very flexible as they rapidly readapt choice after changed conditions (e.g., devaluation of stimuli previously associated with high value). In contrast, habitual choice mechanisms rely on fixed stimulus-response reactions arising after extensive training. Consider again the case of the rat in the T-maze. If it has been rewarded a sufficient number of times for going left, it will tend to choose left again even if there is no reward. Compared to goal-directed mechanisms, habitual mechanisms are less flexible (e.g., they readapt very slowly after devaluation) but also faster and less demanding.

Normative views of animal behavior cast habitual and goal-directed mechanisms of choice in terms of model-free and model-based methods of reinforcement learning (RL), respectively (Daw et al., 2005). Model-free methods use “cached” action values to choose actions (i.e., aggregated values that can be recalled quickly). A long tradition of experimental and theoretical work in neuroscience uses model-free methods of RL, and in particular temporal-difference (TD) methods (Schultz et al., 1997), Q learning (Watkins and Dayan, 1992), and actor-critic architectures (Houk et al., 1995), to explain essential aspects of decision circuits such as dopamine bursts and the functioning of the basal ganglia.

Model-based methods use instead internal forward models to mentally simulate future action possibilities and their associated values. Model-based mechanisms are well known in the reinforcement learning literature (Sutton and Barto, 1981, 1998) and are nowadays increasingly studied in neuroscience and neuroeconomics in relation to perceptual, value-based, and economic choices (Pezzulo et al., 2007; Glimcher et al., 2009; Daw, 2012; Pezzulo and Rigoli, 2011; O’Doherty, 2012; Solway and Botvinick, 2012). Here we focus on goal-directed spatial navigation, which has been linked to the hippocampus – ventral striatum circuit in the rodent brain. It has been reported that rats navigating in mazes stop at decision points and turn the head in one of the possible directions, then to the other. When they turn their heads, place cells in the hippocampus “sweep forward” in the corresponding branch of the maze, as if the rat had really moved in that direction (Johnson and Redish, 2007). In correspondence of forward sweeps, ventral striatum activation is observed as well (van der Meer and Redish, 2009). Based on such evidence, it has been proposed that the hippocampus – ventral striatum circuit implements a *mental simulation* mechanism that realizes goal-directed choice, with the hippocampus linked to forward modeling and the ventral striatum linked to the evaluation of covert expectations of rewards constructed by the hippocampus (van der Meer and Redish, 2009, 2010, 2011; Battaglia et al., 2011; Pennartz et al., 2011; Chersi and Pezzulo, 2012; Erdem and Hasselmo, 2012; Penner and Mizumori, 2012; van der Meer et al., 2012). This view links well with the idea of a “vicarious trial and error” mechanism in rats (Tolman, 1948).

Habitual and goal-directed mechanisms of choice coexist and interact in the brain (Balleine and Dickinson, 1998). However, the proximal mechanisms that are responsible for their interactions are incompletely known. An influential theory proposes a continuous competition between habitual and goal-directed mechanisms of choice (implemented as two separate controllers) regulated by their relative *uncertainty* (Daw et al., 2005; Niv et al., 2006; Dayan, 2009). This theory captures the key role of uncertainty in the arbitration of goal-directed and habitual mechanisms of choice, and can reproduce (among the other things) the effects of *habitization*, or the gradual passage from goal-directed to habitual mechanisms after sufficient learning (Balleine and Dickinson, 1998). Mechanistically, this is due to the fact that the initial uncertainty of the habitual controller (compared to the goal-directed one) is higher (as it learns less efficiently from experience) but becomes lower after sufficient learning. This theory assumes that the model-free and model-based controllers are actively engaged in every decision (although ultimately only one of them is selected) and therefore it cannot explain why the hippocampal forward sweeps, putatively associated with model-based computations, vanish with habitization (van der Meer and Redish, 2009). Furthermore, this theory does not consider that model-based computations might have *costs*, linked to the cognitive effort due to planning (Gershman and Daw, 2011) and to the temporal discounting of rewards due to the time required for planning (Shadmehr, 2010).

We propose that a single instrumental process of decision-making produces both goal-directed and habitual behavior by flexibly combining aspects of model-based and model-free computations. We call this system a *Mixed Instrumental Controller (MIC)*. At decision points, the MIC performs a cost-benefits analysis, comparing the advantage of mental simulation (in terms of improving reward information) with its costs. More specifically, the MIC calculates the *Value of Information* (*VoI*; Howard, 1966) of mental simulation on the basis of uncertainty and of how much the alternative “cached” action values differ against each other. Then, the Value of Information is compared against the cost of mental simulation (in terms of cognitive effort and time). As a consequence of this, goal-directed mechanisms (mental simulations) are activated only when necessary, in line with evidence on rats’ forward sweeps. In sum, the MIC combines model-based and model-free computations and does not lend itself to a complete separation of goal-directed and habitual controllers (in the strict sense devised in Daw et al., 2005); hence the label “mixed.”

**Figure 1:**
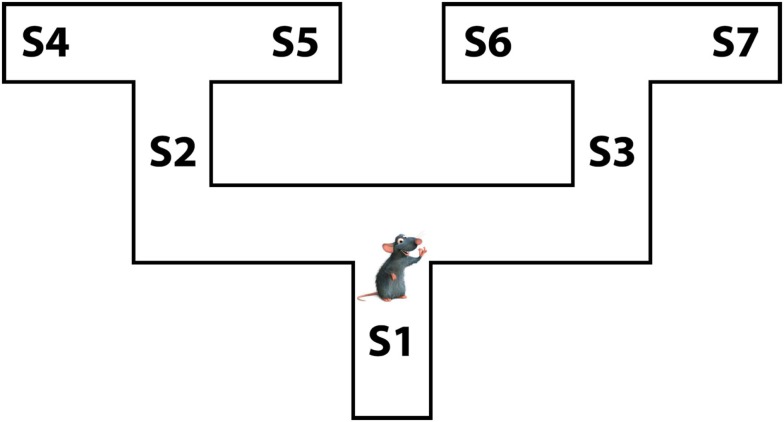
**The rat navigation scenario used in the simulations: a double T-maze**.

In the rest of the article, we introduce the proposed *Mixed Instrumental Controller* model and test it in a simulated rat navigation scenario, in which decisions (going right or left) correspond to the selection of a branch in a double T-maze; see Figure 1. Rewards can be allocated at any of the seven points indicated as *S*1–*S*7. This scenario permits studying how selection of habitual vs. goal-directed processes at decision points changes as a function of learning, and to link elements of the model to neurobiological findings in rodents.

## 2 Methods: The Mixed Instrumental Controller Model

**Figure 2:**
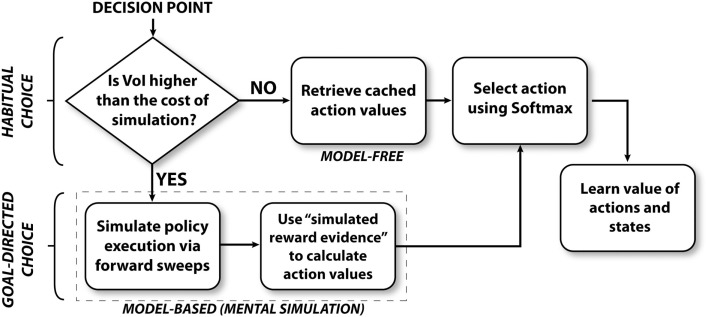
**Overview of the mixed instrumental controller (MIC)**.

Figure 2 illustrates the algorithm followed by the mixed instrumental controller model. This algorithm can be separated in four sub-processes, called meta-choice (between cached values and mental simulation), mental simulation, choice, and learning. Below, we describe each sub-process in details.

### 2.1 Meta-choice between cached values and mental simulation

At decision points (S1, S2, and S3), the agent (a simulated rat) has to decide whether to turn right or left. The agent has stored a prior estimate of each action value (*Q value*, see Watkins and Dayan, 1992), together with an estimate of each Q value uncertainty. Based on this information, at decision points, the agent first chooses whether to mentally explore the action consequences, in order to improve the action value estimates, or to simply rely on prior Q value estimates. This process can be viewed as a meta-choice between habitual (corresponding to “cached” Q values) and goal-directed processes (corresponding to mental simulation). At every decision point, this meta-choice is performed separately for each action (going left and right). In other words, the system might mentally simulate only the more uncertain action(s), not necessarily all.

This meta-choice amounts to computing the *Value of Information* (*VoI*; Howard, 1966) obtained with a mental simulation related to a given action *Act1* (e.g., going left at a decision point when left or right actions are possible). As solving an optimal solution to this problem is generally intractable in non-stationary environments, to determine *VoI*_*Act*1_ we adopt a simpler method described in equation (1):

(1) $$VoI_{\text{Act}1}=\frac{C_{\text{Act}1}}{|Q_{\text{Act}1}-Q_{\text{Act}2}|+\epsilon }$$

This equation indicates that, for each action, our model considers two elements: (1) the difference between the *Q*_*Act*1_ value and the *Q*_*Act*2_ value of the alternative action (plus an ϵ to ensure that the sum is non-zero); (2) the uncertainty (*C*_*Act*1_) relative to *Q*_*Act*1_. The ratio between the two elements represents the estimated *VoI*_*Act*1_ obtained with mental simulation. This value is compared with the *cost* of mental simulation, which can be thought to be connected to the cognitive effort due to search (Gershman and Daw, 2011) and the temporal discounting of rewards due to the passing of time (Shadmehr, 2010). This cost is implemented here as a fixed threshold *γ*.

### 2.2 Mental simulation

When *VoI*_*Act*1_ is smaller than the threshold *γ*, the agent relies on the cached *Q*_*Act*1_ value estimates for choice. On the contrary, when *VoI*_*Act*1_ is bigger than the threshold, forward sweeps are performed to simulate the effects of possible action executions. These simulated effects are then considered as pseudo-observations and are used to improve the estimation of *Q*_*Act*1_.

**Figure 3:**
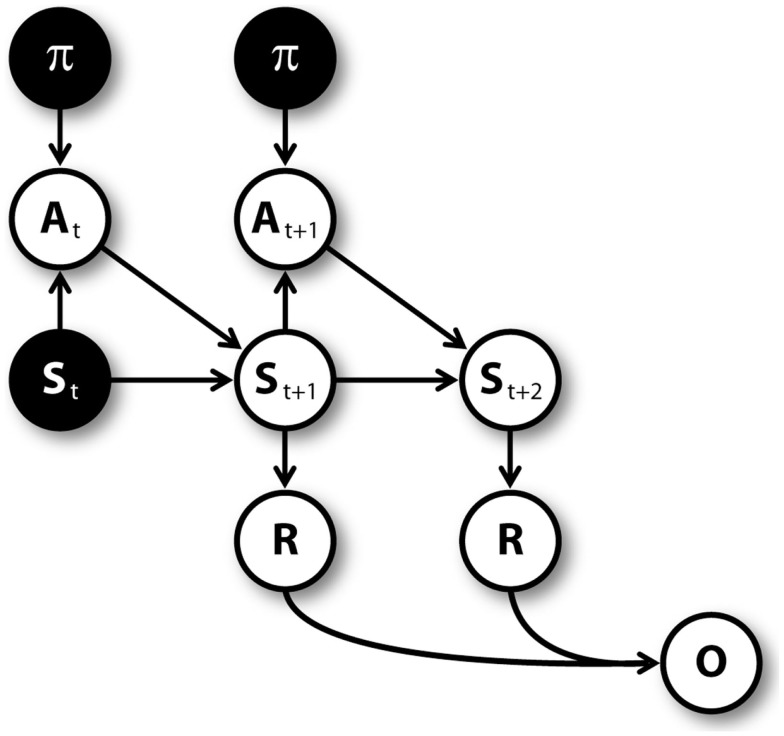
**Graphical model for mental simulations, unrolled for three time steps**. Filled nodes are “clamped” (i.e., considered as observed) during mental simulation (see Table 1).

Figure 3 shows the graphical model (Dynamic Bayesian Network; Murphy, 2002) used for mental simulation (see Botvinick and An, 2008; Dindo et al., 2011; Pezzulo and Rigoli, 2011; Solway and Botvinick, 2012 for related models). Nodes represent random variables including policies (*π*), actions (A), belief states (S), rewards (R), pseudo-observations (O) along with their temporal index *t*. Arrows connecting nodes indicate conditional probabilities among corresponding variables. Mental simulation consists in “clamping” current state and policy nodes (in other words, in considering these nodes as observed), and compute the conditional aggregated “value,” which depends on the rewards gained at every time steps. The clamped policy at the first time step corresponds to the simulated action, while the policies clamped at following time steps are randomly chosen with equal probability. For instance, at S1 the agent could simulate the “going left” action by clamping the policy of going left at the first time step, and clamping a random policy (e.g., going right) at the second time step.

Mental simulations are repeated for several times, and every time the computed value is stored. The number of simulations is proportional to uncertainty (*C*_*Act*1_); the proportion is regulated by a parameter *λ*. In addition, the number of simulated time steps for every simulation depends on uncertainty as well. Specifically, when uncertainty is higher than a threshold *ζ*, the agent simulates a sequence of actions (i.e., a whole path in the T-maze) and uses rewards to compute its aggregated value. Alternatively, the agent simulates a shorter path (whose length is regulated by a parameter *η*) and retrieves the Q value of one of the actions associated to the last simulated state. This Q value incorporates the cumulative expected value from that state on, rather than only the value of the state (i.e., it is a *return* and not a *reward* in reinforcement learning, see Sutton and Barto, 1998). Values relative to future states are discounted with a factor *δ*.

Once all mental simulations have been executed, the computed values are considered as pseudo-observations (*O*_1_, *O*_2_, …, *O_n_*, one for each simulation) and are used to improve the estimate of *Q*_*Act*1_. The stored value is used as a prior$\left(Q_{Act1}^{Prior}\right)$ and the pseudo-observations are used to compute a posterior value $\left(Q_{Act1}^{Posterior}\right)$. This computation is described by equation (2) (assuming that the distribution variance of the *Q*_*Act*1_ value is known and is equal to 1, see Bishop, 2006):

(2) $$Q_{Act1}^{Posterior}=\frac{Q_{Act1}^{Prior}+C_{Act1}\cdot \sum_{i=1}^{N}\;O_{i}}{1+C_{Act1}\cdot N}$$

where *C*_*Act*1_ is the uncertainty, namely the prior variance on the mean of the $Q_{Act1}^{Prior}$ value distribution, *O_i_* is the pseudo-observation *i*, and N is the number of pseudo-observations.

### 2.3 Choice

At every decision point, a choice between actions is made by considering the value of the different possible actions (*Q*_*Act*1_ and *Q*_*Act*2_). Note that this value can be either the cached Q value (if mental simulation was not used) or the posterior Q value calculated with equation (2) (if mental simulation was used). The choice is made according to the following softmax equation:

(3) $$\begin{array}{rcll} & P\left(Action=Act1|Q_{Act1},Q_{Act2}\right) & & \\ & \;=\frac{exp\left(\beta \cdot Q_{Act1}\right)}{exp\left(\beta \cdot Q_{Act1}\right)+exp\left(\beta \cdot Q_{Act2}\right)} \end{array}$$

where *Q*_*Act*1_ and *Q*_*Act*2_ are the Q values relative to the two possible actions (say going left or right at a decision point), and *β* is the inverse temperature parameter.

### 2.4 Learning

The MIC has two forms of learning.

#### 2.4.1 On-line learning of C and Q values

**Figure 4:**
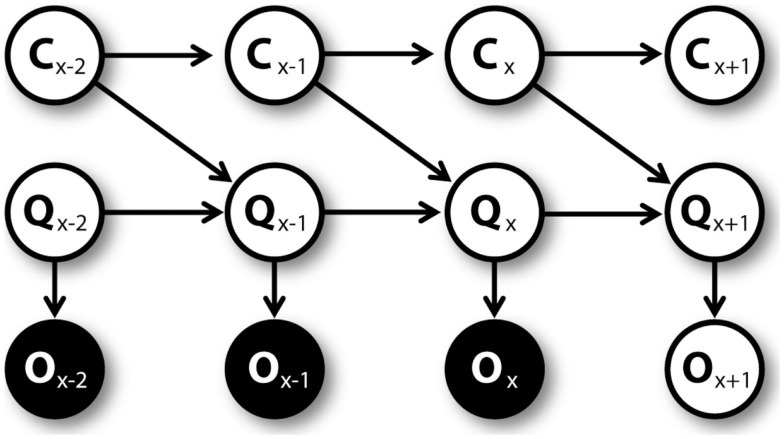
**Graphical model for learning *C* and *Q* values, unrolled**.

Once the agent executes an action, he moves toward a new position and, in some cases, collects a reward. On the base of this novel experience, the agent learns. First, the *Q*_*Act*1_ value corresponding to the executed action is updated. The obtained reward, which is summed up to the Q value corresponding to the best action associated to the new position, is considered as an observation O. This observation is used to estimate the Q value at the following trial using the generative model represented by the graphical model shown in Figure 4. At every trial *x*, the prior *Q*_*Act*1,*x*_ value and uncertainty *C*_*Act*1,*x*_ are used by a particle filtering algorithm to compute the *Q*_*Act*1,*x*+1_ value and the uncertainty *C*_*Act*1,*x*+1_ at trial *x* + 1. The prior *Q*_*Act*1,*x*_ value considered here is the “cached” *Q*_*Act*1_ value that is available before mental simulations (if any) were made.

The specific particle filtering algorithm is the following: for n = 1 to N, random vectors [*C*_*Act*1,*n*_*Q*_*Act*1,*n*_] are sampled from the prior Gaussian distributions of uncertainty *N* ∼ (*C*_*Act*1,*x*_, *k*) (where *k* is a known parameter) and of Q value *N* ∼ (*Q*_*Act*1,*x*_, *C*_*Act*1,*n*_). Then, the sampled vectors are weighted proportionally to *P*(*O*_*Act*1,*x*_/*Q*_*Act*1,*n*_). After this, N vector samples are drawn from the previous vector set, each with a probability proportional to its weight. Finally, the posterior uncertainty is computed as *C*_*Act*1,*x*+1_ = ∑*C*_*Act*1,*n*_/*N* and the posterior Q value as *Q*_*Act*1,*x*+1_ = ∑*Q*_*Act*1,*n*_/*N*.

#### 2.4.2 Value learning

The model uses a model-based method to learn state values (i.e., the rewards *R* in the graphical model shown in Figure 3). Every time a reward is encountered in a state *s*, the mean of the expected reward conditional to that state *R*(*s*)_*x*+1_ is updated according to equation (4):

(4) $$R{\left(s\right)}_{t+1}=R{\left(s\right)}_{t}+\alpha \left(R_{observed}-{R\left(s\right)}_{t}\right)$$

where *α* is a learning rate.

## 3 Results of the Simulations

We tested the MIC model in five simulated experiments. In the simulations, an artificial agent faced a double T-maze (see Figure 1) and, for several trials, had to choose twice to go either right or left. The simulations tested two key predictions of the model. First, we expected that the MIC model was able to learn the correct policy based on available rewards. Second, we expected that the MIC model executed forward sweeps only in certain circumstances, namely when the *VoI* was high. Specifically, we expected to observe forward sweeps at the beginning of learning in all simulations. In addition, forward sweeps were expected to gradually decrease and disappear in simulations where variances were small and/or alternative Q values were not close to each other (simulations 1, 2, 4), contrary to simulation 5 where variances were high and alternative Q values were close to each other. Finally, we expected forward sweeps to reappear following unexpected changes in reward (simulation 3), and to decrease and disappear again as learning proceeded. In all the simulations, we assumed that the agent already knew the transition function, namely the conditional probabilities of outcomes given previous states and actions in the graphical model shown in Figure 3. The parameters and constants used in all the simulations are shown in Table 2.

**Table 1:** **Nodes of the graphical model of Figure 3**.

Node	Explanation	Values
*π*	Policies	{S1 → left, S2 → left, S3 → left} … {S1 → right, S2 → right, S3 → right}
A	Actions	Left, right (or equivalently: Act1, Act2)
R	Rewards	[0 … n]
S	Belief states	S1, S2, S3, S4, S5, S6, S7
O	Pseudo-observations	[0 … n]

**Table 2:** **Parameters and constants used in all the simulations**.

Label	Explanation	Value
*α*	Learning rate for the model-based value representations	0.2
*β*	Inverse temperature parameter of the softmax function	0.4
*γ*	Threshold for mental simulation	0.5
–	Discount factor	1
ϵ	Small number used in the VoI to avoid division by zero	0.0001
*ζ*	Threshold relative to uncertainty for shortening the mental simulation	3
*η*	Length of the simulation when uncertainty is lower than *ζ*	1
–	Starting reward values for the model-based representations	1
–	Initial value of uncertainty in the simulations	4
κ	Uncertainty variance	1
*λ*	Number of forward sweeps during mental simulation	C × 3
–	Prior Q values at the first trial	1

### 3.1 Simulation 1: Simple and stable environment with low variance

In the first simulation, a reward having a mean of 5 (r = 5) was placed at S7 (i.e., top right), while other positions had zero mean reward. Reward variance was relatively small for all positions, namely 0.2. The aim of this experiment was studying the gradual transition from goal-directed to habitual mechanisms of choice as a function of learning. Indeed, in stable environments, a given sequence of actions (in this case, right-right) is always reinforced and, after a certain amount of learning, can be selected by using habitual mechanisms, without the effort entailed by mental simulations. We hypothesized, as experience increased, a decrease in number and length of mental simulations (corresponding to goal-directed control), leading to relying on prior Q estimates (corresponding to habitual control).

**Figure 5:**
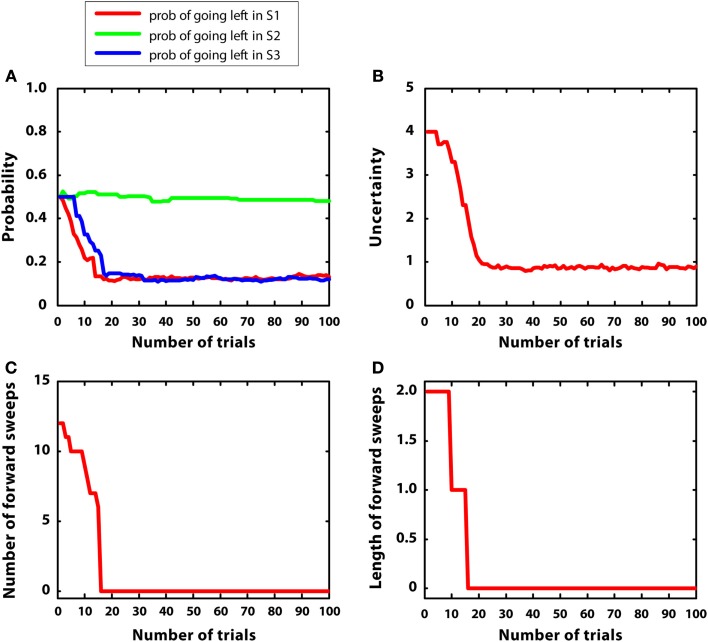
**Results of simulation 1, simple and stable environment with low variance, for 100 trials**. **(A)** (top left) Shows the probability of going left at S1, S2, and S3 during the trials. **(B)** (top right) Plots the uncertainty for going right at S1. **(C)** (bottom left) Shows the number of forward sweeps (associated to mental simulation) used for the choice at S1; zero means that mental simulation is not used. **(D)** (bottom right) Shows the length of forward sweeps used for the choice at S1.

Figure 5 describes the experimental results. Figure 5A shows the probability of choosing left turns at S1, S2, and S3. It shows a rapid decrease of preference for left turns at S1 and S3, as it was expected given that reward could be collected with two right turns. Turning right or left at S2 was equiprobable as neither S4 nor S5 were rewarded. Figure 5B shows the value of uncertainty along trials for going right at S1, which diminished rapidly. Figure 5C shows the number of samples used for the mental simulation for going right at S1, which is proportional to uncertainty. A value of zero indicates that the mental simulation is not used at all. Our results show that, during learning, mental simulations decreased in number, suggesting a gradual shift from goal-directed to habitual control. Moreover, Figure 5D indicates that, along learning, the length of forward sweeps decreased as well. The mechanisms tested in the present simulation can explain why learning in stable and simple environments produces habitization, which parallels a reduction (in number and length) of hippocampal forward sweeps and covert expectation of reward in ventral striatum (van der Meer and Redish, 2009). The development of habits entails also a “shift” of activation in dorsolateral striatum from actual reward locations to decision points and then to starting points (Jog et al., 1999). In our framework, this corresponds to the states in which the agent is highly confident of acquiring reward (i.e., at S7 before learning, at S3, and successively at S1 after learning).

### 3.2 Simulation 2: Complex and stable environment with low variance

In the second simulation, multiple rewards were placed in the maze: S2 (r = 2), S4(r = 1), S7 (r = 5). Like in the previous simulation, reward variances were relatively small (0.2). The goal of this simulation was to test whether the agent was able to shift from goal-directed to habitual control in a more complex environment.

**Figure 6:**
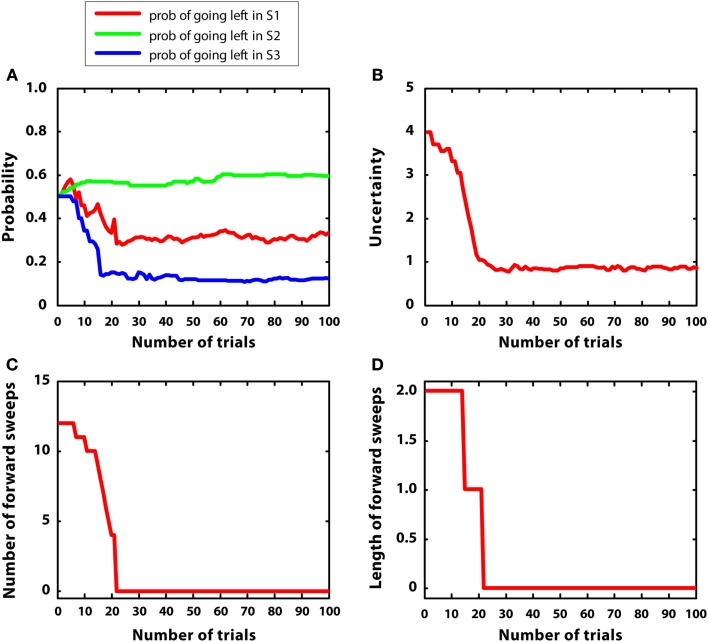
**Results of simulation 2, complex and stable environment with low variance. (A–D)**: see Figure 5

Figure 6 describes the results. Figure 6A indicates that the agent was able to learn the correct policy. Figure 6B shows a decrease in uncertainty along learning for the action “going right” at S1. Figures 6C,D indicate that both the number and length of forward sweeps diminished along learning. The results of this simulation show that the MIC model can choose adaptively even in environments that have multiple rewards. In addition, due to the low reward variance, the model habituated (i.e., diminished forward sweeps) almost as fast as simulation 1. Compared to simulation 1, the choice of actions was more variable, matching the amount of rewards at different branches of the T-maze. This is due to the use of a softmax rule, which selects actions in proportion to their Q values rather than always selecting the action having the highest Q value.

### 3.3 Simulation 3: Non-Stationary environment

In the third simulation, a single reward (r = 5) was initially placed at S7, and then moved to S4 after 50 trials. Reward variances were relatively small (0.2). The aim of this simulation was studying how the model re-adapts to novel contingencies. In other words, the agent had to learn an action sequence (right twice) and, after contingencies had changed, to re-learn a novel action sequence (left twice).

**Figure 7:**
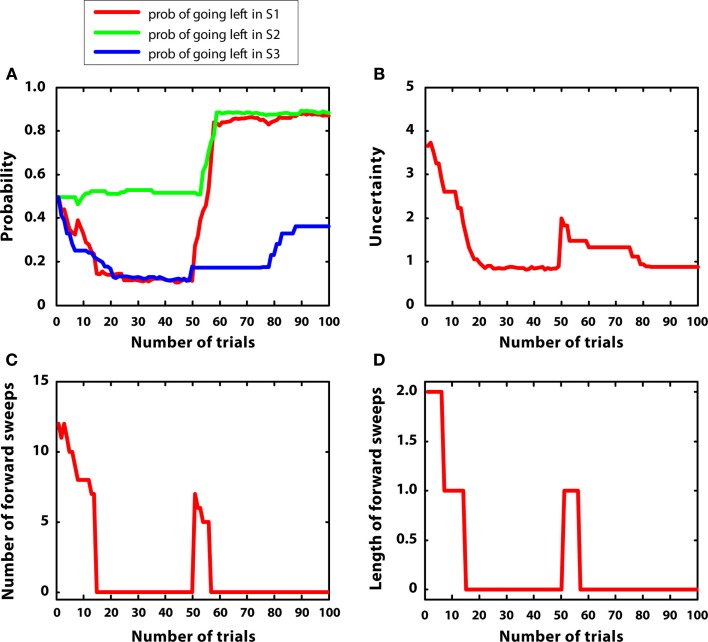
**Results of simulation 3, non-stationary environment. (A–D)**: see Figure 5

Figure 7 describes the results. Figure 7A shows that the policy was updated correctly in correspondence with the introduction of novel contingencies (Balleine and Dickinson, 1998). Figure 7B indicates that uncertainty decreased from trial 1 to 50, but, at this point, it increased again because previous contingencies had changed. This pattern was mirrored by the number and length of forward sweeps, shown in Figures 7C,D. These results show that the habitual system takes control in stationary environments but, after surprising outcomes are encountered, goal-directed mechanisms (corresponding to mental simulations) are activated again, due to a rapid uncertainty increase. This pattern of results suggests a specific prediction done by the MIC model in relation to the mechanisms regulating forward sweeps in rats, which requires empirical testing.

### 3.4 Simulation 4: Simple environment with high variance

**Figure 8:**
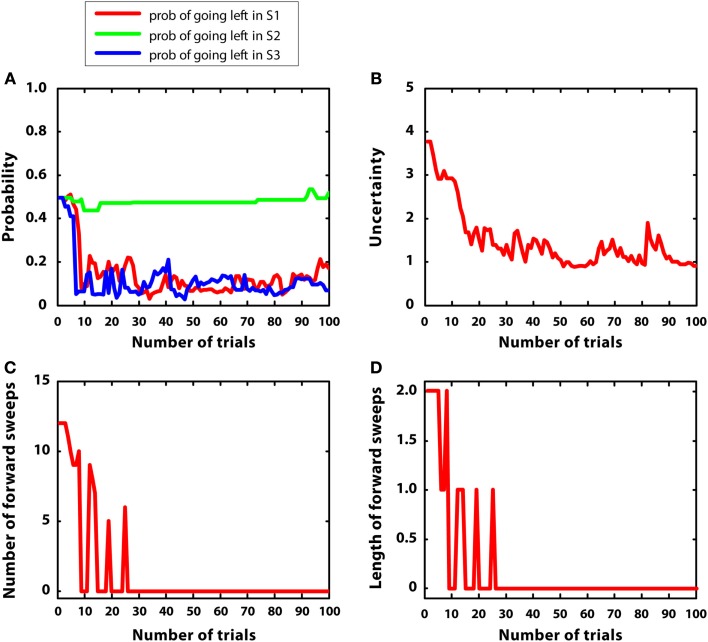
**Results of simulation 4, simple environment with high variance. (A–D)**: see Figure 5

In this simulation, mean rewards were as in simulation 1 (i.e., r = 5 at S7), but with 5-times larger variances (var = 1). We hypothesized that uncertainty was bigger and less stable in this condition, compared to simulation 1. Figure 8 describes the results. Figure 8A shows that the agent learned the correct policy (although beliefs were less stable compared to simulation 1). Figure 8B confirms that uncertainty was bigger and less stable than simulation 1. Figures 8C,D shows that, at the beginning of learning, mental simulations were activated for more trials compared to simulation 1. This is consistent with the idea that forward sweeps in the hippocampus are not only a function of experience (i.e., the more experience, the less forward sweeps) but also a function of environmental uncertainty (Gupta et al., 2010). However, with a certain amount of learning, in this simulation the habitual system took control as in simulation 1, and forward sweeps were no more activated. The reason was that, although variance was high, the environment was “simple.” In other words, the difference between alternative Q values was big and the animal was quite confident about the best choice to take. This pattern of results represents a specific prediction of the MIC model, which requires empirical testing.

### 3.5 Simulation 5: Complex environment with high variance

**Figure 9:**
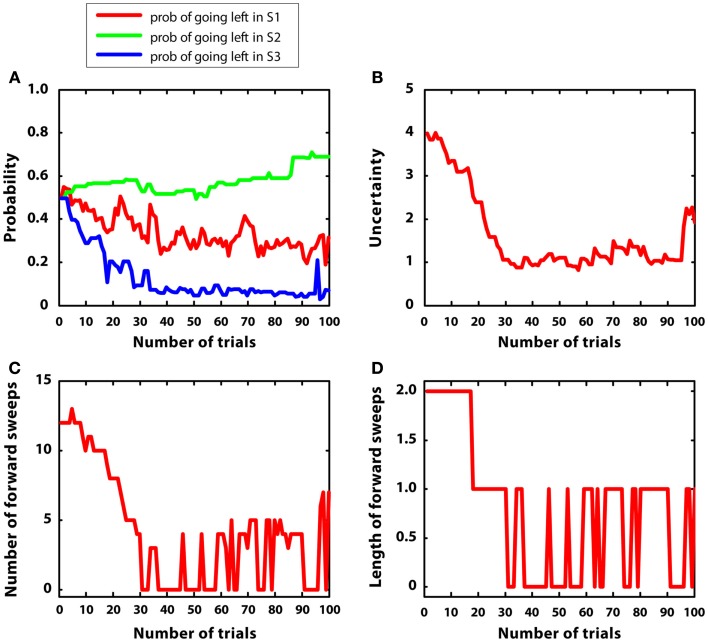
**Results of simulation 5, complex environment with high variance. (A–D)**: see Figure 5

In the last simulation, mean rewards were like simulation 2 (i.e., r = 2 in S2; r = 1 in S4, r = 5 in S7); however, in this case, reward variances were bigger, namely they were equal to 1. The goal of this simulation was to observe the artificial agent in a complex environment with high variance. Figure 9 describes the results. Figure 9A confirms that the agent was able to learn the correct policy, although beliefs were more noisy than in simulation 2. Figure 9B shows that uncertainty was bigger and less stable than in simulation 2. This led to activate mental simulations along the whole learning period (see Figures 9C,D) although to a larger extent at the beginning. The use of mental simulations along the whole learning period is caused by two factors. First, high reward variance increased uncertainty. Second, in this simulation, the environment was complex, namely different paths were not much different to each other in terms of total reward. Indeed, going left at S1 led to r = 3, whereas going right led to r = 5, which are relatively close to each other. These results suggest that in complex and uncertain environments the forward sweeps could persist for a longer time, and the passage from goal-directed to habitual strategies could be incomplete.

## 4 General Discussion

The Mixed Instrumental Controller (MIC) is an integrative model describing how model-based (mental simulation) and model-free mechanisms (Q learning) could interact in both cooperative and competitive ways, producing a continuum of habitual and goal-directed strategies of choice.

In the Mixed Instrumental Controller, model-free mechanisms are used by default and supported by model-based computations when the Value of Information of the latter surpasses its costs; this is typically true when uncertainty is high and alternative cached action values are close to each other. Furthermore, the relative contribution of model-based mechanisms can vary: the less the uncertainty, the fewer the samples used to implement the forward sweeps. In sum, the MIC permits to flexibly balance model-free and model-based methods depending on environmental circumstances.

To decide when mental simulation is necessary, the Mixed Instrumental Controller solves a “dilemma” that is similar to the well known *exploration-exploitation dilemma*, except that in this context the exploration is “mental” and not overt. Specifically, the *mental exploration* consists in performing mental simulations to access expectancies and associated reward predictions, and ultimately to better estimate action values. The *exploitation* consists in choosing an action on the basis of the already available (“cached”) estimate of action values, rather than performing mental simulation. The dilemma can be solved by comparing the Value of Information that can be retrieved using mental simulation with the cost of the simulation. Computing an optimal solution to this problem is generally intractable in non-stationary environments, and it is still unclear if and how the brain does so (Aston-Jones and Cohen, 2005; Daw et al., 2006; Pezzulo and Couyoumdjian, 2006; Behrens et al., 2007; Cohen et al., 2007; Bromberg-Martin and Hikosaka, 2011; Niv and Chan, 2011). The MIC model implements an approximate solution to this problem that considers accuracy of choice (i.e., probability of acquiring higher reward) and uses a fixed cost of acquiring information (in terms of cognitive effort and time); the former factor favoring mental exploration, and the latter exploitation. Overt exploration is not explicitly modeled in the MIC, but it results from the adoption of a softmax function for the choice.

Our simulations in environments having different characteristics (stable or volatile, low or high variance) show that there are multiple factors that can cause the Value of Information to be higher, and most notably the variance and the difference in value between the competing alternatives. Generally, mental simulations at decision points diminish after sufficient learning, in line with evidence showing that in this condition habitization replaces goal-directed mechanisms of choice (Jog et al., 1999). However, if variance is high or if the values of the alternatives are too close, the system is slower in developing habits. Different from alternative models, in the MIC the habitization is accompanied by a reduced use of model-based computations; this mechanism can explain why hippocampal-striatal forward sweeps, possibly encoding covert simulations at decision points, vanish after sufficient experience (van der Meer and Redish, 2009).

When environmental contingencies change, mental simulations are used anew, consistent with evidence of a passage from habitual to goal-directed strategies after outcome devaluation (unless it occurs after “overtraining”). When contingencies change, the goal-directed system can immediately change behavior. Furthermore, changed environmental conditions increase the VoI and speeds up the updating of C and Q values; see Figure 4. However, the reaction to outcome devaluation can be slower (or impaired) when actions are over-trained (Dickinson, 1985) because the (non-active) goal-directed system cannot instruct an immediate change of strategy and updating C and Q values takes longer.

It is worth noting that although the MIC model is sensible to the volatility of the environment, this element is not explicitly modeled (but, see below for a possible extension of the model). Finally, our results in the more complex environments (with high uncertainty and variance) are in keeping with evidence that forward sweeps are not a simple replay of previous experience but are modulated by task uncertainty (Gupta et al., 2010). Moreover, the MIC model makes the further prediction that the difference between alternative cached action values has a role as well in influencing forward sweeps.

## 5 Conclusion

We proposed that essential aspects of goal-directed and habitual control can be captured within a single instrumental process of decision-making, the Mixed Instrumental Controller (MIC), which flexibly balances and combines model-based and model-free computations. We linked the functioning of the MIC model to a neural circuit formed by the hippocampus and the ventral striatum, which has been shown to be active during goal-directed navigation and the choice between spatially defined goals.

The MIC model elaborates on a previous influential model (Daw et al., 2005; Niv et al., 2006; Dayan, 2009) which emphasizes that goal-directed and habitual mechanisms of choice are linked to model-based and model-free methods of reinforcement learning, respectively, and which assigns a key role to uncertainty. At the same time, the MIC departs from this model in that it assumes that model-based calculations are only used when the Value of Information they can furnish is higher than their costs. Another distinguishing point is the fact that while in the previous theory model-free and model-based processes produce two competing instrumental controllers, in the MIC they act in concert. First, although generally mental simulations are used to retrieve the rewards associated to future states, they can also retrieve Q values that permit to aggregate the value of several time steps, as it is typical of model-free algorithms. Combining these two methods (for instance, performing forward search until a reliable cached value is available) is typical in game playing set-ups (Baum and Smith, 1997) and understanding how the brain might do so is an important avenue for future research (Glascher et al., 2010; Simon and Daw, 2011a). Second, model-free and model-based processes provide complementary information to calculate action values. This is evident if one considers that, in equation (2), the cached Q value is used as a prior and updated using model-based calculations.

Another peculiarity of our model is the way mental simulation is realized. In the MIC, mental simulation is computationally implemented as a *sequential sampling* procedure using the graphical model described in Figure 3. The method we adopt consists in “clamping” one policy at a time (see Solway and Botvinick, 2012), which produces a serial process of (simulated) internal experience sampling. This method is different from the idea of a “tree search” as it is typically described in normative approaches (Niv et al., 2006), and from models of parallel “diffusion” processes for planning (Ivey et al., 2011). It produces a serial forward search that better captures the nature of forward sweeps in the hippocampus (see also Lengyel and Dayan, 2008; Bornstein and Daw, 2011). Furthermore, the specific algorithm used for the forward search, i.e., particle filtering, produces a (noisy) accumulation of evidence about rewards, which links well to sequential sampling dynamics used for perceptual decisions and memory search (Ratcliff, 1978) and the “ramping” activity of primate neurons during choice (typically, in the neuronal areas that control the effectors used for the choice; Shadlen and Newsome, 2001; Cisek and Kalaska, 2005; Ding and Gold, 2010). Overall, then, our mental simulation system describes the value-based computations of the hippocampus – ventral striatum circuit in terms that are analogous to those of perceptual-based decisions, and are coherent with the idea of “decision by sampling” (Stewart et al., 2006).

All these characteristics distinguish the MIC from the model of Daw et al. (2005) and from several others, which we shortly review below. Similar to the MIC, it has been recently proposed that model-based computations are activated only when the Value of Information they add is bigger than the cost of waiting they entail (Keramati et al., 2011). Similar to the MIC model, the Value of Information is computed by considering the uncertainty and the distance between alternative action values; however, different from the MIC model the model-based component is expected to have perfect information. The major difference between the model of Keramati et al. (2011) and the MIC is that how model-based computations are performed and used. Indeed, the former model shifts completely from habitual to goal-directed control when the Value of Information is sufficiently high. Conversely, the latter model integrates “cached” values and model-based estimation, and thus results in a “mixed” control. In addition, in the MIC model model-based computations are performed using a serial sampling process; the samples vary in number and length and model-based computations can be performed only for a sub-set of available actions. These features have been adopted to fit better with the evidence available on rats’ forward sweeps, which are thought to correspond to model-based computations.

The aforementioned models (Daw et al., 2005; Keramati et al., 2011) and others (Simon and Daw, 2011b) assume that model-based and model-free methods can only compete, not cooperate. The DYNA model is one of the few systems in which model-based and model-free methods cooperate (Sutton, 1990). In DYNA, only the habitual system is responsible for making decisions, but the goal-directed system can train it by providing off-line predictions. A recent study uses the DYNA system to explain the shift between habitual and goal-directed systems and retrospective revaluation (Gershman et al., 2012). In the MIC model mental simulations are used on-line, during the choice, and are responsible for the forward sweeps in the hippocampus at decision points. Below we discuss a straightforward extension of the MIC model that uses mental simulations both on-line and off-line.

An alternative view of the memory consolidation process is that it consists in a *chunking* of action sequences. In this view, model-free methods are not used: all actions are first executed in a model-based way and then gradually chunked and transformed into habits (Dezfouli and Balleine, 2012). Different from this theory, the MIC uses both model-free and model-based methods, and describes the transition from goal-directed to habitual behavior in terms of changed Value of Information rather than chunking.

### 5.1 Future improvements of the MIC model

There are several aspects of the MIC model that can be further elaborated. First, the MIC currently uses simplified methods to calculate Value of Information and the costs of simulation. The method we devised has several limitations; for instance, it does not consider the absolute value of the actions but only their relative values, and only uses a fixed threshold. The current formal analyses of Value of Information take some of these aspects into consideration but are computationally impractical; furthermore, it is unclear how they link to neural computations (Howard, 1966). As our knowledge of how the brain addresses these problems increases, better methods can be devised that permit to quantify the costs and benefits of mental exploration, and to realize a better cost-benefits analysis.

The proposed model can be easily extended by permitting the model-based part to train the model-free part off-line and in absence of overt behavior, similar to other RL algorithms such as DYNA (Sutton, 1990) and prioritized sweeping (Moore and Atkeson, 1993). The values of C and Q can be updated even when the agent is not acting by endogenously steering mental simulations to produce “fake” reward observations O, and then using the same learning methods as described in sec. 2.4. With this straightforward extension the MIC can benefit from both on-line and off-line mental simulations using the same mechanisms. We chose not to use off-line mental simulations in our experiments because in the scenarios we simulated there could be too little time to complete the off-line training within experimental trials (otherwise we would never observe forward sweeps at decision points). Rather, we hypothesize that off-line training could have a more prominent role when there is enough time for memory consolidation (e.g., during pauses and sleep, but also when there is enough time between experimental trials). In the proposed “extended” version of the MIC model, mental simulations support both decision-making (when used on-line) and memory consolidation (when used off-line). Indeed, there are various demonstrations that the rat hippocampus replays (forward and backward) sequences of neural activity experienced during overt behavior both when the animal pauses (and is awake) and when it is asleep (Foster and Wilson, 2006; Diba and Buzski, 2007; Koene and Hasselmo, 2008; Peyrache et al., 2009; Gupta et al., 2010; Carr et al., 2011); still the behavioral significance of these findings is disputed. Some studies emphasize the importance of forward sweeps for decision-making (van der Meer and Redish, 2009), while other studies highlight the consolidation of recent memories into long-term memory and the formation of “cognitive maps” of the environment (Tolman, 1948; O’Keefe and Dostrovsky, 1971; Morris et al., 1982). We hypothesize that these apparently distinct views can be reconciled if one considers the aforementioned distinction between on-line and off-line uses of mental simulations in the MIC model. It is worth noting that the precise mechanisms regulating off-line mental simulations remain to be established. Off-line training could be regulated by similar principles of optimization as in the meta-choice we described. For example, the agent could simulate being at a decision point, decide whether or not to activate the model-based component using the Value of Information computations of equation (1), and use the particle filtering algorithm of sec. 2.4 for training the habitual system. Alternatively, it could eschew the Value of Information computations and only consider the accuracy of the habitual system (e.g., the variance of Q values) or more simply try to systematically update all the Q values. The plausibility of these and other hypotheses remains to be established.

The proposed model can also be improved by explicitly modeling environmental volatility. The MIC is implicitly sensible to volatility and changed reward contingencies. However, it is plausible that living organisms explicitly model volatility (Behrens et al., 2007; Kepecs et al., 2008). In turn, an estimate of volatility permits to better regulate the Value of Information (as in volatile environment uncertainty cannot be reduced using mental simulation), to adjust learning rates adaptively, and to modulate the rate of overt exploration (which is at the moment sidestepped using a parameterized *softmax* function). A related issue is considering the quality of the internal model and the controllability of the environment when choosing a controller; computational modeling studies suggest that it might be favorable to select closed-loop methods in well-modeled regions and open-loop methods in regions that are not (or cannot) be modeled with high accuracy (Kolter et al., 2010).

Another important direction for future studies is devising biologically plausible and scalable algorithms to implement the proposed model-based computations. At the moment, model-based methods are computationally prohibitive for large state spaces, but progresses on sampling methods (Doucet et al., 2000) and Monte Carlo search (Silver and Veness, 2010) are encouraging. Not only these methods are interesting from a computational viewpoint, but they could also shed light on how mental simulations and forward planning are mechanistically implemented in the brain, as suggested by recent studies that link brain activity with probabilistic computations (Ma et al., 2006; Doya et al., 2007) and sampling methods (Fiser et al., 2010; Berkes et al., 2011).

Furthermore, the MIC uses model-based computations and mental simulations for action selection and learning, but it leaves unspecified if they can be also used for other purposes. An intriguing proposal is that mental simulations can be used to monitor actions initiated by the habitual system until their successful completion (Alexander and Brown, 2011). This would permit a rapid initiation of action, and also its subsequent revision if mental simulation uncovers negative consequences that the habitual system did not take into consideration. It is worth noting that this mechanism could be another way how model-free and model-based methods cooperate.

We have linked the model-based computations of the MIC to a neural circuit formed by the hippocampus and the ventral striatum. The reason for our choice is that this circuit has been linked to goal-directed computations in spatial navigation (i.e., the scenario that we chose to exemplify the characteristics of the MIC). However, it is plausible that the brain uses additional (or different) neuronal circuits for model-based computations outside the spatial domain. We hypothesize that the MIC captures essential principles of instrumental control that are not restricted to goal-directed spatial navigation; however, understanding if the model-based computations of the MIC apply to instrumental choice at large remains an open objective for future research.

A further aspect to consider is how the MIC architecture could potentially include Pavlovian mechanisms. In relation to this, two possibilities should be considered. Pavlovian processes might substantially act in parallel with instrumental ones. Alternatively, Pavlovian and instrumental representations might largely overlap. Although contrasting findings have been reported, evidence suggests that Pavlovian and goal-directed values are segregated functionally and neurally. For instance, following devaluation, Pavlovian effects, contrary to goal-directed ones, are visible even without incentive learning. Moreover, lesions of different portions of amygdala, ventromedial prefrontal cortex, and striatum, have differential impact on Pavlovian and goal-directed mechanisms (Balleine and O’Doherty, 2009). Overall, this evidence suggests that Pavlovian and instrumental mechanisms work in parallel (see also Rigoli et al., 2012), and future implementations of the MIC should consider this fact.

Finally, the MIC model is currently limited in that it only considers one level of granularity of actions and states. In contrast, the control of behavior has been recently linked to hierarchical reinforcement learning models (Botvinick, 2008; Botvinick et al., 2009; Frank and Badre, 2012), in which actions can be specified at different levels of abstractness and temporal extension (see also Verschure et al., 2003). Extending the MIC with hierarchical action organization would provide extra flexibility, allowing it, for example, to select and plan actions at more abstract levels, and to connect with the growing literature on prefrontal control hierarchies (Fuster, 1997; Koechlin and Summerfield, 2007; Wise, 2008).

### 5.2 Real-Time dynamics and putative neuronal architecture of the mixed instrumental controller

The MIC model offers a computational-level explanation of the interactions between habitual and goal-directed processes of choice in the context of spatial navigation. While the real-time dynamics of mental simulation are explicitly modeled using the particle filtering algorithm, the moment-by-moment dynamics of the action selection process are sidestepped using the process model described in Figure 2. Below we discuss how the MIC model could implement real-time dynamics of choice through a neural architecture.

We take as our starting point the *affordance competition hypothesis* (Cisek and Kalaska, 2010): a parallel model of decision-making that describes choice as a dynamic competition between two (or more) action alternatives (say, go left or right). In the affordance competition hypothesis, multiple plans for action are formed in parallel and compete over time until one has sufficient support to win the competition. In terms of the MIC, the default habitual processes (plausibly including mappings between stimuli and motor representations) mediate this selection by instructing previously reinforced stimulus-response associations. In this architecture, response dynamics correspond to the activity of neuronal populations in frontoparietal cortex, forming a sort of motor map for the potential responses (Cisek, 2006), whose selection is plausibly supported by the basal ganglia (Redgrave et al., 1999; Chersi et al., 2012; Lepora and Gurney, 2012). In the context of spatial navigation and the choice between spatially defined goals, the hippocampus is also involved to support (among the other things) spatial representation and processing. During the choice, the presence of an appropriate stimulus (say, the sight of a branch of the T-maze) could produce a strong peak of activation in the motor map in correspondence of the to-be-selected action. However, this is only effective when the stimulus-response associations are strong enough (e.g., after habitization). When the potential action plans have little support (e.g., before sufficient learning), or when the choice is highly uncertain, the motor map could encode several low-intensity and high variance peaks of activation. In these cases, cognitive control and monitoring mechanisms could inhibit action execution and allow for more information to be collected via model-based computations, until confidence is high or the costs of acquiring it surpasses the benefits.

In the MIC, there is not a univocal value representation, but different aspects of valuation correspond to different parts of the model; this is consistent with recent theories that recognize the contribution of different brain areas to utility representation and processing (Ito and Doya, 2011; Pennartz et al., 2011). State values (and reward expectancies) could be associated to ventral striatum (Lansink et al., 2009; van der Meer and Redish, 2009), ventrotegmental area, basolateral amygdala, and orbitofrontal cortex (Padoa-Schioppa and Assad, 2006; Yin et al., 2008; McDannald et al., 2012). In our model, state values correspond to *S* → *R* transitions; in a previous work we have also shown how these values can be modulated by the agent’s internal motivational state (Pezzulo and Rigoli, 2011). Dorsolateral striatum could encode cached action values and could have a role in encoding uncertainty (Yin et al., 2004; Kepecs et al., 2008; represented in our model by *Q* and *C*, respectively). It is worth noting that although the dorsal/ventral division of the striatum (which we also re-propose here) has been associated to segregated habitual and goal-directed controllers, respectively, our model does not necessarily imply a complete segregation, but is compatible with the view that the controllers could partially overlap. The mapping of specific parts of the striatum with different computations (model-based and model-free) and modes of control (goal-directed and habitual) is still controversial (see Bornstein and Daw, 2011).

The MIC is consistent with the idea that the ventral striatum supports model-based reward representations (activated during forward sweeps), as suggested by van der Meer and Redish (2010). This idea is distinct from the standard view that the ventral striatum plays the role of “critic” in actor-critic RL theories, and is recruited exclusively during learning (Houk et al., 1995). However, the MIC is consistent also with an alternative possibility, coherent with the ventral striatal role as “critic.” It is indeed possible that this structure encodes the “fictive” prediction error which, in the MIC model, is used to update prior Q “cached” values with pseudo-observations produced by mental simulation. This hypothesis generates the specific prediction that the signal in ventral striatum correlates with the “fictive” prediction error (i.e., with the discrepancy between “cached” and goal-directed values) rather than with goal-directed values. By using devaluation, for example, it could be possible to test these alternative hypotheses.

Another aspect of the MIC model is relative to the meta-choice, the calculation of the Value of Information, and the cognitive control of the computations. A relatively simple form of cognitive control has been linked to *optimal stopping* problems, in which it is necessary to consider the confidence of actions and the cost to be late before taking an action (Gold and Shadlen, 2001, 2007). It has been argued that optimal stopping and more sophisticated forms of meta-choice could be based on mechanisms for monitoring, uncertainty consideration and behavioral inhibition. With these mechanisms, the architectures for action specification and selection described before can become able of goal-directed choice and cognitive control, consistent with the view that these more advanced abilities could derive from elaborations of brain designs that solve simpler sensorimotor processes (Pezzulo, 2008, 2011; Pezzulo and Castelfranchi, 2009; Cisek and Kalaska, 2010; Cisek, 2012). In the MIC, these mechanisms could improve the choice by permitting model-based mechanisms to support or even substitute the default habitual control mode. In the current implementation, this is done by mentally simulating and collecting covert expectations of reward and goals, but anatomical considerations point also to more sophisticated mechanisms such as mental time travel and the construction of novel episodic memories (Schacter et al., 2007, 2012; Buckner, 2010). Although the neural underpinnings of the control architecture are incompletely known, we speculate that monitoring processes in the anterior cingulate cortex could signal the opportunity to overcome stimulus-bound responses (Botvinick et al., 2001; Alexander and Brown, 2011), the Value of Information computations could reuse cached action, and uncertainty values, and the passage from stimulus-bound to internally generated (simulated) contexts necessary for the model-based computations could be linked to rostral prefrontal cortex (Burgess et al., 2007).

These and other aspects of brain implementations of goal-directedness remain open objectives for future research. Indeed, our study is part of a large initiative investigating model-based decision-making in the brain (Balleine and Dickinson, 1998; Daw et al., 2005; Dayan, 2009; Green et al., 2010; Rao, 2010; Daw, 2012; Pezzulo and Rigoli, 2011; Simon and Daw, 2011b; Solway and Botvinick, 2012). Model-free RL methods have provided useful insights to study the neural neurobiology of action values and habitual behavior. Analogously, model-based RL mechanisms could help studying the neural underpinnings of mental simulations, outcome predictions, and goal-directed choice (O’Doherty, 2012). It is important to consider that there are many possible variants of model-based RL methods (as there are multiple forms of model-free RL computations), possibly linking to different neural substrates (Daw, 2012). So, it remains to be evaluated what computational proposals better capture the brain’s ability to flexibly choose and act in a goal-directed manner.

## Conflict of Interest Statement

The authors declare that the research was conducted in the absence of any commercial or financial relationships that could be construed as a potential conflict of interest.
